# Isolation, identification, and characterization of *Listeria* spp. from various animal origin foods

**DOI:** 10.14202/vetworld.2015.695-701

**Published:** 2015-06-06

**Authors:** Deepti N. Nayak, C. V. Savalia, I. H. Kalyani, Rajeev Kumar, D. P. Kshirsagar

**Affiliations:** 1 Department of Veterinary Public Health and Epidemiology, Vanbandhu College of Veterinary Science and Animal Husbandry, Navsari Agricultural University, Navsari - 396 450, Gujarat, India; 2 Department of Veterinary Microbiology, Vanbandhu College of Veterinary Science and Animal Husbandry, Navsari Agricultural University, Navsari - 396 450, Gujarat, India

**Keywords:** animal origin foods, *Listeria monocytogenes*, *Listeria* spp, polymerase chain reaction, virulence genes

## Abstract

**Aim:**

The present study was undertaken with the prime objective of isolating and identifying *Listeria* spp. from various foods of animal origin sold at retail market outlets in the city of Navsari, Gujarat.

**Materials and Methods:**

Total 200 samples comprising of milk, milk products, meat, and fish (50 each) collected aseptically from local market which were subjected first to pre-enrichment in half strength Fraser broth followed by enrichment in full strength Fraser broth and subsequent plating on PALCAM agar. The growth with the typical colony characteristics were further identified up to species level on the basis of their morphological and biochemical characteristics. Cultures identified as *Listeria monocytogenes* were further subjected to *in vitro* pathogenicity tests and detection of different virulence-associated genes *viz. act*A, *hly*A, and *iap* using polymerase chain reaction.

**Results:**

Of the total 200 food samples of animal origin; 18 (9%) were found positive for *Listeria* spp. which were identified as *Listeria seeligeri* (6, 33.3%), *Listeria innocua* (5, 27.7%), *Listeria welshimeri* (4, 22.2%), and *L. monocytogenes* (3, 16.6%). The highest prevalence was observed in milk samples (8). Species wise, 6 isolates of *L. seeligeri* which included two each from cow milk, buffalo milk, and meat samples; 5 L. *innocua* isolates included four recovered from fish and one from meat sample; 4 L. *welshimeri* comprised of two isolates from ice cream and one each from buffalo milk and meat sample; and 3 isolates of *L. monocytogenes* recovered from milk (1 cow and 2 buffalo milk). All 3 L. *monocytogenes* isolates screened for the presence of virulence genes *viz*. *act*A, *hly*A, and *iap* using the specific primers revealed the presence of all the genes suggesting the possibility of danger of foodborne listeriosis among raw milk consumers.

**Conclusion:**

*Listeria* spp. was isolated from 9% (18/200) of the animal origin food samples *viz*.; milk, milk products, meat, and fish with the highest prevalence in the milk samples. *L. monocytogenes* was isolated from 3 milk samples only. *L. seeligeri* was the predominant species isolated followed by *L. innocua, L. welshimeri, and L. monocytogenes* in this study. *L. monocytogenes* were found to carry virulence genes like *actA*, *hly A*, and *iap* genes suggesting the pathogenic potential of these isolates.

## Introduction

The organisms of genus *Listeria* are Gram-positive, facultative anaerobic, non-spore-forming, rod-shaped bacteria with a low G + C content. The genus consists of different species *viz*.; *Listeria monocytogenes*, *Listeria ivanovii*, *Listeria* *seeligeri*, *Listeria innocua*, *Listeria welshimeri, Listeria grayi*, *Listeria marthii*, *Listeria rocourtiae*, as well as newly described non-pathogenic *Listeria aquatica* spp. nov., *Listeria floridensis* spp. nov., *Listeria cornellensis* spp. nov., *Listeria grandensis* spp. nov., and *Listeria riparia* spp. nov. [1-3]. *L. monocytogenes* is a primary human pathogen amidst rare reports of illnesses caused by *L. seeligeri*, *L. ivanovii*, and *L. innocua* [4].

*L. monocytogenes* causes gastroenteritis varying from mild to severe illness reported in veterinarians, farmers and abattoir workers; and circling disease which is a manifestation of basilar meningitis besides spontaneous abortions in animals. The pathogen is transmitted via fecal-oral route directly from animals to humans. Vertical transmission from mother to neonate occurs transplacentally or through an infected birth canal. The fatality ranges from 30% to 75% especially in high risk groups like pregnant women, unborn or newly delivered infants, and the elderly people as well as persons with severe underlying disease conditions like immune-suppression, AIDS, chronic conditions like cirrhosis [5,6]. This organism is halotolerant, and can grow and multiply at refrigeration temperatures [7].

Though not phenomenal, the number of human *Listeriosis* cases in India, have been in the rise with reports on sporadic cases and incidence in clinical samples and has been quoted as an emerging foodborne disease in India by Chugh [8]. Similarly, reports have also been made by Aurora *et al*. [9,10] on the incidence of *L. monocytogenes* in milk based ready-to-eat foods from Agra region.

Two of the most widely-used culture reference methods for detection of *Listeria* in all foods are the FDA Bacteriological and Analytical Method (BAM) [11] and the International Organization for Standardization method [12]. Molecular approaches for DNA isolation and identification such as the polymerase chain reaction (PCR) have shown to be faster and more reliable than conventional techniques.

Various foods have been implicated in the spread of *L. monocytogenes* repeatedly found in raw milk, soft cheese, and pasteurized dairy products including ice cream, fish and fish products, and ready-to eat foods [13-15]. Taking into consideration all these aspects, this study was conducted to study the prevalence of *Listeria* spp. in foods of animal origin *viz*.; milk, milk products, meat, and fish samples.

## Materials and Methods

### Ethical approval

Ethical approval is not required to pursue this type of study.

### Sample collection

A total of 200 food samples, comprising of milk (50), ice cream (20), milkshake (15), fruit salad (15), and 50 samples each of fish and meat were collected from randomly selected retail shops of Navsari city. Almost all the ice cream shops from different areas were covered, milk samples were collected from tabelas as well as vendors and fish and meat samples were collected from vendors as well as market. The samples were collected only once from each place. Samples were collected in icebox and stored at 4°C till further analysis attempted in the Department of Veterinary Public Health and Epidemiology, Vanbandhu Veterinary College, Navsari Agricultural University, Navsari.

### Standard strains

The standard strains of *L. monocytogenes* (MTCC 1143)*, Rhodococcus equi* (MTCC 1135), and *Staphylococcus aureus* (MTCC 3160) used in the study were procured from the Microbial Type Culture Collection and Gene Bank, Institute of Microbial Technology, Chandigarh (IMTC), India.

### Isolation and identification of Listeria spp.

ISO 11290 method was employed to isolate the organisms, whereby pre-enrichment of 25 g sample was done in 225 ml half strength Fraser broth containing selective supplements (HiMedia) for 24 h at 30°C, which was followed by second enrichment of 0.1 ml of pre-enriched Fraser broth content in 10 ml full strength Fraser broth containing selective supplements (HiMedia) for 48 h at 37°C incubation temperature. After the enrichment procedure, the inoculum was plated on PALCAM agar (HiMedia) and incubated for 48 h at 37°C.

The gray-green colonies surrounded by diffuse black zone on PALCAM agar were picked up and further purified on Tryptone Soya Yeast Extract agar (TSYEA). Subsequently, pinpoint colonies of TSYEA were subjected to identification procedures which included Gram’s staining followed by a microscopic examination, catalase test, and oxidase test. The characteristic Gram-positive, coccobacillary or short rod-shaped organisms which were catalase positive and oxidase negative, were sub-cultured in Brain heart infusion (BHI) broth at 25°C for 12-18 h. Subsequently, the cultures showing typical tumbling motility were considered as “presumptive” listeria isolates, which were in turn subjected to detailed biochemical tests *viz*.; methyl red, Voges-Proskauer, nitrate, and sugar fermentation tests with xylose, rhamnose, mannitol, and α-methyl D-mannopyranoside as described in Table-1.

**Table-1:** Biochemical test pattern and *in-vitro* pathogenicity profile of the *Listeria* spp.

Species identified	Biochemical tests					Sugar fermentation pattern				Hemolysis on 5% SBA	CAMP test with *S. aureus* (S) and *R. equi* (R)
	
	C	O	MR	VP	Ni	L-Rh	D-Xy	αMdm	D-Ma		
*L. monocytogenes*	+	-	+	+	-	+	-	+	-	+	+(S)
*L. seeligeri*	+	-	+	+	-	-	-	-	-	+	-
*L. welshimeri*	+	-	+	+	-	+	+	+	-	-	-
*L. innocua*	+	-	+	+	-	+	-	+	-	-	-

C=Catalase, O=Oxidase, Ni=Nitrate, L-Rh=Rhamnose, D-Xy=Xylose, αMdm=α-Methyl-d-mannoside, D-Ma=Mannitol, *L. monocytogenes=Listeria monocytogenes, L. seeligeri=Listeria seeligeri, L. welshimeri=Listeria welshimeri, L. innocua=Listeria innocua*, SBA=Sheep blood agar, CAMP=Christie Atkins Munch Peterson, MR=Methyl red, VP=Voges Proskauer

### In vitro pathogenicity tests

The isolates were tested for the type and the degree of hemolysis on 5% sheep blood agar and the Christie Atkins Munch Peterson (CAMP) test was conducted using *Listeria* isolates, *S. aureus*, and *R. equi* standard strains to observe for hemolysis between *Listerial* strain and the *S. aureus* or *R. equi* owing to the synergistic effect of their hemolysins in case of a CAMP-positive reaction as indicated in Table-1.

### DNA extraction protocol

The DNA of the *L. monocytogenes* was extracted using the protocol of QAIGEN DNeasy blood and tissue kit with minor modification in centrifugation parameters that is doubling the time of centrifugation. A loop full of culture grown overnight in 4 ml BHI broth at 37°C was used to isolate the DNA.

### Detection of virulent genes

The *L*. *monocytogenes* isolates were screened for the presence or absence of virulence-associated gene(s) by using the standard PCR protocols for the detection of *act*A, *hly* A, and *iap* genes as per the methodology described previously [15,16]. Primers used for PCR reaction are mentioned in Table-2 [15-19]. The PCR protocol was standardized using *L. monocytogenes* (MTCC 1143). Initial denaturation was carried out at 95°C for 2 min followed by denaturation 95°C for 15 s followed by annealing 60°C for 30 s and extension at 72°C for 1 min 30 s and repeated for 35 cycles.

**Table-2:** Primers used for PCR reaction.

Gene	Primer sequence	Base pairs	Reference
*act*A	F: CGC CGC GGA AAT TAA AAA AAG R: ACG AAG GAA CCG GGC TGC TAG	839	[15]
*hly*A	F: GCA GTT GCA AGC GCT TGG AGT GAA R: GCA ACG TAT CCT CCA GAG TGA TCG	456	[16,18,19]
*iap*	F: ACA AGC TGC ACC TGT TGC AG R: TGA CAG CGT GTG TAG TAG CA	131	[17]

PCR=Polymerase chain reaction

### Agarose gel electrophoresis protocol

For PCR amplification, 5 μl of the PCR products was mixed with 1 μl of ×6 gel loading buffer and electrophoresed along with DNA molecular weight marker (3B BlackBio Biotech) on 2.0% agarose gel containing ethidium bromide (@ 0.5 μg/ml) at 5 V/cm for 60 min in 0.5 × tris-borate-ethylenediaminetetraacetic acid buffer. The amplified product was visualized as a single compact band of expected size under UV light and documented by gel documentation system (SynGene, Gene Genius BioImaging System, UK).

## Result

Of 200 samples, total 18 (9%) food samples were found positive for *Listeria* spp. The food sample wise, maximum prevalence was noticed in milk samples (8/50, 16%) followed by 8% (4/50) each, of meat and fish samples, and 4% in milk products (2/50). The species wise spread was expressed as maximum 6 cultures (3%) of *L. seeligeri* followed in descending rate of occurrence as 5 (2.5%) of *L. innocua*, 4 (2%), *L. welshimeri*, and 3 (1.5%) isolates of *L. monocytogenes*.

Of 6 L. *seeligeri* isolates, two each were recovered from cow as well as buffalo milk (8% on type of milk basis), and 2 (4%) from meat samples. Of total 5 isolates of *L. innocua*, 4 (8%) were cultured from fish samples and 1 (2%) from the meat. *L. welshimeri* (4) isolated from 2 of 20 (10%) ice cream samples and one each, from 25 buffalo milk (4%) and 50 meat (2%) samples. A potent pathogen *L. monocytogenes* was isolated from 3 milk samples (1.5%) only, which included 2 (8%) buffalo milk and 1 (4%) cow milk samples as described in Table-3.

**Table-3:** Prevalence of *Listeria* spp. in various food samples of animal origin.

Type of sample	Number of samples analyzed	Number of samples positive for *Listeria* spp. (%)				Total positive samples

		*L. monocytogenes*	*L. seeligeri*	*L. innocua*	*L. welshimeri*	
Cow milk	25	1 (4)	2 (8)	0	0	3 (12)
Buffalo milk	25	2 (8)	2 (8)	0	1 (4)	5 (20)
Total	M=50	3/50 (6)	4/50 (8)	0	1/50 (2)	8/50 (16)
Ice cream	20	0	0	0	2 (10)	2 (10)
Milkshake	15	0	0	0	0	0
Fruit salad	15	0	0	0	0	0
Total	MP=50	0	0	0	2/50 (4)	2/50 (4)
Meat	50	0	2 (4)	1 (2)	1 (2)	4 (8)
Fish	50	0	0	4 (8)	0	4 (8)
Total	200	3 (1.5)	6 (3)	5 (2.5)	4 (2)	18 (9)

M=Milk, MP=Milk product, *L. monocytogenes=Listeria monocytogenes, L. seeligeri=Listeria seeligeri, L. welshimeri=Listeria welshimeri, L. innocua=Listeria innocua*

**Figure-1:**
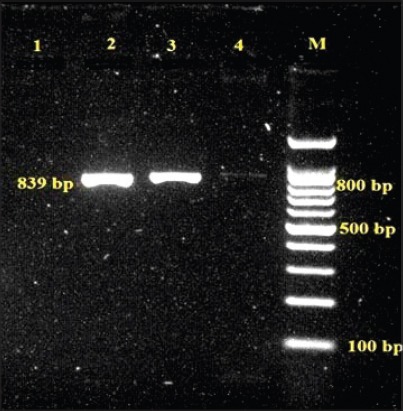
Agarose gel showing polymerase chain reaction amplified product of 839 bp for *act*A gene in *L. monocytogenes* isolates, Lane 1: Negative control, Lane 2: Positive control, Lane 3-4: Samples positive for *act*A, Lane M: 100 bp DNA ladder.

**Figure-2:**
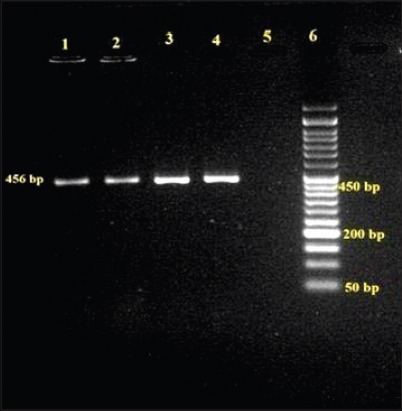
Agarose gel showing polymerase chain reaction amplified product of 456 bp for *hly*A gene in *L. monocytogenes* isolates, Lane 1-3: Samples positive for *hly*A, Lane 4: Positive control, Lane 5: Negative control, Lane 6: 50 bp DNA ladder.

**Figure-3:**
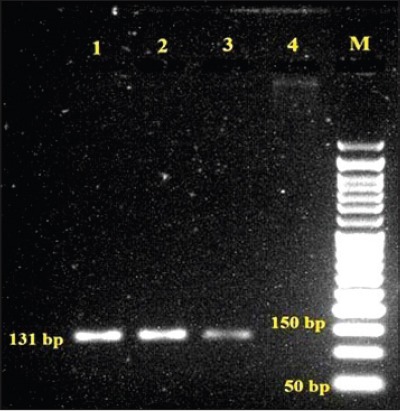
Agarose gel showing polymerase chain reaction amplified product of 131 bp for *iap* gene in *L. monocytogenes* isolates, Lane 1-2: Samples positive for *iap*, Lane 3: Positive control, Lane 4: Negative control, Lane M: 50 bp DNA ladder.

All three *L. monocytogenes* isolates cultured from animal origin food samples, which were subjected for detection of virulence-associated gene studies by PCR yielded desired amplification of 839 bp of *act*A (Figure-1), 456 bp of *hly*A (Figure-2) and 131 bp of *iap* (Figure-3) genes, respectively.

## Discussion

### Overall prevalence

The findings of overall prevalence of 9% *Listeria* spp. were relatively lower than the earlier findings *viz.;* 12.5% from 530 samples of dairy products, meat and RTE foods [20], 16% of 1481 food samples [21], 38.1% of 134 food samples [22]. These values are higher than those observed in our study might be due to variation in the place of work.

In the present study, *L. monocytogenes* was isolated from 3 (1.5%) food samples only, none other than milk (3/50, 6%). Earlier studies reported occurrence of this pathogen in varying levels on overall basis, *viz*.; 17.5 % among 320 samples of different vegetables, meat, sea foods, and dairy products [22], 7.9% of 1537 beef, pork, chicken, fish, raw milk, ice cream samples [23] and 3.5% (8/225) milk, cheese, chicken, and red meat specimens [24] which is quite higher than present findings. This variation in the findings may be due to disparity in the type of foods considered in the present work, and number of samples analyzed.

### Sample wise prevalence

According to the type of milk, 12% (3/25) cow milk and 20% (5/25) buffalo milk samples contained *Listeria* spp., with an overall prevalence of 16% in 50 milk samples. Compared to the present study quite higher prevalence of *Listeria* spp. in milk was observed as 21.32% [25], 22% [26], 26.13% [27], and 54.7% [28]. Although source of the bacteria the milk samples in the present work is obscure, work reviewed indicated that *Listeria* spp. do isolated from rotting vegetation, soil, and water. In contrast, lower level of prevalence was reported in the earlier observations made by Yoshida *et al.*, [29] as 0.3% by Bhilegaonkar *et al*. as 5.78% [30], by Gulhan *et al*. as 6% [31], by Kalorey *et al*. as 6.75% [32], by Moura *et al*. [33] as 6.8%, and by Alzubaidy *et al*. [24] who observed 9% incidence of *Listeria* spp. in raw milk samples, indicative of lower level of contamination, might be due to good hygienic practices in the places from where samples drawn. The prevalence of *L. monocytogenes* detected from milk samples in this study was 6% which is in congruence to 5.78% [30] prevalence of *L. monocytogenes* in farm and bulk milk samples as well as with 6.25% milk samples out of 64 analyzed by Barbuddhe *et al*. [27]. The prevalence values are also comparable to 6.5% obtained from 861 milk samples [34] as well as 5.88% prevalence of *L. monocytogenes* reported from raw milk samples by Waghmare [25] but lower than 19.6% observed by Molla *et al*. [35]. Out of 137 milk samples analyzed by Sarangi *et al*. [36] four (2.91%) *L. monocytogenes*, three (2.18%) *L. innocua*, and two (1.45%) *L. welshimeri* were isolated which is lower than that observed in the present study.

Examination of 50 milk product samples comprising of 20 ice cream, 15 milkshake, and 15 fruit salad samples revealed the presence of *Listeria* spp. in 10% ice cream samples only identified as *L. welshimeri*. The prevalence of *Listeria* spp. in ice cream, i.e. 4% in the present study was much lower as compared to those observed by various workers as 43.5% out of 137 ice cream samples analyzed [33], 41.7% *Listeria* spp. in ice cream from 134 food samples analyzed [14], 16.7% in ice-cream from 290 samples of traditional milk products in Iran [13] thereby demanding care in preparation of the milk product to protect health of consumers. Comparatively lower degrees of incidence have been recorded in ice cream samples *viz*.; 5% [37], 6% [38] and 6.1% [23], respectively; which is indicative of marginally safe food hygiene practices might have followed at places under the studies.

The present study indicated that out of 50 meat samples surveyed, 8% (4/50) samples yielded growth of *Listeria* spp. The findings are in approximation with earlier reports of Barbuddhe *et al*. [27] as 10.17% of 167 meat samples positive for *Listeria* spp. as well as 6.7% positivity of Listeriae among 150 meat samples screened denoted by Nayak *et al*. [39]. Values of prevalence of *Listeria* spp. in meat samples obtained as 50% [14], 27.2% [20] and 22% [24] were higher than the present study provided warning signal for safeguarding human health as far as usage of meat as food is concerned. None of 50 meat samples examined during the present investigation yielded *L. monocytogenes*.

All the four fish isolates recovered in the present investigation were identified as *L. innocua*. Literature reviewed showed comparatively high prevalence in seafood samples, *viz*.; by Murtiningsih and Sunarya [40] who tested 124 fish and seafood samples, wherein *L. innocua* was identified as dominant species cultured from 11.3% samples, 17.5% prevalence of *L. innocua* and *L. monocytogenes* reported in Mangalore India [22], 32.3% samples out of 324 tropical seafood and environmental samples positive for *Listeria* spp. with *L. innocua* as most prevalent bacteria of fisheries from Kerala, India [41] are in resemblance to the present study. But in contrast, *L. innocua* was isolated from 0.9% samples of the 300 marine food samples analyzed by Momtaz *et al*. [42]. The prevalence of *Listeria* spp. in the present investigation turned out to be 8% expressed on the basis of 50 fish samples surveyed, which is in agreement with finding of Swetha *et al*. [43] who found 8% level of isolation of *Listeria* spp. in 60 fish samples from Hyderabad, India.

However, higher values than present work were detected during studies conducted by Ghazi M. Jabir Al-Maliki [44] and Wang *et al*. [45] who isolated *Listeria* spp. from 13 and 13.8% fish and seafood samples, respectively. On the other hand, values lower than the present study were also observed as 1.2% from shellfish [23] and 5% dry fish [41].

A desired amplified product of 839 bp similar to the reference strain, sequenced for *act*A gene was present in all the *L. monocytogenes* isolates. Earlier studies also cited *act*A gene in *L. monocytogenes* recovered from fish and marine food samples [16,41,42]. Only two of the three isolates identified as *L. monocytogenes* in the present work amplified 131 bp product in lieu to the reference strain with the use of primer pair synthesized for *iap* gene which is in parallel to the findings of Jallewar *et al*. [16] wherein *iap* gene was detected in *L. monocytogenes* as well as *L. seeligeri* from fish samples, from ice-cream samples [36], from fish samples [41], and from milk, water, and human clinical samples [46]. All the 3 isolates yielded desired amplified product of approximately 456 bp, similar to the reference strain of *L. monocytogenes* using the primer pair for *hly*A which encodes pore-forming listeriolysin O necessary for bacterial escape from the phagosomes of host cells into the host cytosol. The characterization of *L. monocytogenes* was studied and isolates were confirmed by PCR amplification of *hly* virulence gene from various foods [23], fish samples [16,41], and milk samples [32].

## Conclusions

*Listeria* spp. was isolated from 9% (18/200) of the samples of animal origin foods which included milk, milk products, meat, and fish with highest prevalence from milk samples. *L. monocytogenes* was isolated from 3 milk samples only. Among all *L. seeligeri* was the predominant species isolated followed by *L. innocua, L. welshimeri, and L. monocytogenes* in this study. All three *L. monocytogenes* isolates showed the presence of *act*A and *hly*A whereas only two isolates amplified *iap* virulence genes.

## Authors’ Contributions

CVS designed and monitored the study. DNN and DPK collected references, samples, and performed laboratory investigation. CVS, IHK, DNN, and RK drafted and revised the manuscript. All authors read and approved the final manuscript.
